# MicroRNA-210 Protects PC-12 Cells Against Hypoxia-Induced Injury by Targeting BNIP3

**DOI:** 10.3389/fncel.2017.00285

**Published:** 2017-09-22

**Authors:** Yonggang Luan, Xiaoli Zhang, Yongli Zhang, Yubin Dong

**Affiliations:** Department of Neonatal Intensive Care Unit, Zhoukou Central Hospital Zhoukou, China

**Keywords:** microRNA-210, neonatal brain injury, hypoxic injury, BNIP3, PI3K/AKT/mTOR signal pathway

## Abstract

MicroRNA (miR)-210 is the most consistently and predominantly up-regulated miR in response to hypoxia in multiple cancer cells. The roles of miR-210 in rat adrenal gland pheochromocytoma (PC-12) cells remain unknown. We aimed to explore the possible effect of miR-210 in neonatal brain injury. We explored the potential molecular mechanism by using PC-12 cells under hypoxia. Scramble miRs, miR-210 mimic, miR-210 inhibitor or its negative control were respectively transfected into PC-12 cells. Cell viability, migration, invasion and apoptosis were also assessed to evaluate hypoxia-induced cell injury. The expression level of miR-210 was identified by quantitative real-time polymerase chain reaction (qRT-PCR) analysis. Apoptosis-related protein expression as well as key kinases in the phosphatidylinositol 3-kinase (PI3K)/AKT/mammalian target of rapamycin (mTOR) signal pathway was studied by Western blot analysis. Hypoxia suppressed cell viability, migration and invasion, but promoted apoptosis through activation of mitochondrial- and caspase-dependent pathways. Hypoxia markedly induced up-regulation of miR-210 in PC-12 cells. Overexpression of miR-210 protected PC-12 cells against hypoxia-induced injury. Bcl-2 adenovirus E1B 19 kDa-interacting protein 3 (BNIP3) was proven to be a target gene of miR-210 in PC-12 cells. miR-210 overexpression ameliorated the hypoxia-induced injury in PC-12 cells by down-regulating BNIP3. Hypoxia-induced alterations of key kinases in the PI3K/AKT/mTOR signal pathway were affected by aberrant expression of BNIP3. These findings suggested that miR-210 protected PC-12 cells against hypoxia-induced injury by targeting BNIP3, involving the PI3K/AKT/mTOR signal pathway.

## Introduction

Neonatal brain injury is a type of non-progressive brain damage; there are several contributing factors including congenital cerebral dysgenesis, cerebral palsy, infantile severe illness and nervous system dysfunction caused by trauma (du Plessis and Volpe, 2002; Hagberg, 2004; Ma and Zhang, 2017). As the term “neonatal brain injury” suggests, it mainly occurs in the perinatal period and preterm infants (Ma and Zhang, 2017). This injury is associated with severe morbidity and mortality rates where 50% of newborns die or have permanent neurological deficits (Fotopoulos et al., 2001). In developing countries, the incidence rate of neonatal brain injury was almost up to 10-fold higher (Lawn et al., 2005). Moderate to severe neonatal brain injury occurs at a rate of 0.1%–0.2% of full-term live births, along with a total incidence of 0.3%–0.5% (Whitelaw and Thoresen, 2002; Shankaran and Laptook, 2007; Gonzalez and Ferriero, 2008). According to previous studies, there are three main contributing factors, including hypoxia-induced ischemia, brain injury caused by bacterial or viral infection and brain damage caused by accidents, that are responsible for progression of neonatal brain injury (Qiu et al., 2013). For better understanding of the disease pathogenesis and also for better management of neonatal brain injury, elucidation of the underlying molecular mechanism will be of great significance.

It is a major cause of perinatal hypoxic ischemic (HI) injury when the oxygen supply falls below the normal levels, also termed hypoxia, which plays a crucial role in the pathogenesis of perinatal HI injury (Bacon and Harris, 2004). The molecular mechanisms underlying the brain ischemia, during which the blood flow to brain is insufficient, are extremely complex. Mounting evidence has reported that hypoxia inducible factor-1α (HIF)-1α is essential for the developments of normal brain and injuries (Gruber and Simon, 2006; Kim et al., 2006; Gordan and Simon, 2007). Recently, microRNAs (miRs) are demonstrated to be critical in brain ischemic injury. miRs are non-coding RNAs that could repress gene expression through binding to the 3′-untranslated region (3′UTR) of transcripts, resulting in degradation of transcript or translational inhibition of the target genes. Among them, miR-210 is a master miR of the hypoxia response, belonging to a specific group of miRs termed “Hypoxamirs” which are regulated by hypoxia (Ma and Zhang, 2017). miR-210 has been implicated in neural cell death and pathogenesis of neonatal HI brain injury in a recent study by Ma et al. (2016). The study demonstrated that HI led to significant increase of miR-210 expression in neonatal rat brains. Moreover, the HI-induced neuronal death was decreased and long-term neurobehavioral function recovery was improved by the down-regulation of miR-210, supporting a detrimental role of miR-210 in neonatal HI brain injury (Ma et al., 2016).

Bcl-2 adenovirus E1B 19 kDa-interacting protein 3 (BNIP3), identified first by using the adenovirus E1B-19K protein as bait in a yeast two-hybrid screen, is a hypoxia-inducible death protein (Boyd et al., 1994; Gang et al., 2015). BNIP3 acts as an essential mediator of cell survival and programmed cell death (Swiderek et al., 2013). The previous literature has reported the potential interactions between miR-210 and BNIP3 in neural progenitor cells (NPCs; Wang et al., 2013). Bioinformatics methods also identified the putative complementary connection between miR-210 and BNIP3. Thus, elucidating the precise details between miR-210 and BNIP3 are of great importance for the study of neuroprotection. In addition, the phosphatidylinositol 3-kinase (PI3K)/AKT/mammalian target of rapamycin (mTOR) pathway is widely accepted to be a central hub for modulation of cell proliferation, cycle and apoptosis (Manfredi et al., 2015). Hypoxia-induced apoptosis in rat pheochromocytoma (PC-12) cells can be repressed by HIF-1α mutant (P402A and P564A)-modified bone marrow stem cells partially through the PI3K/AKT pathway (Zhong et al., 2012). Therefore, BNIP3 and the PI3K/AKT/mTOR pathway could be related to HI injury.

In this study, we aimed to investigate the possible roles of miR-210 in neonatal brain injury using an *in vitro* cell model with hypoxia injury, and to explore the potential molecular mechanism, involving BNIP3 and the PI3K/AKT/mTOR pathways, in PC-12 cells. Our study supports a novel therapeutic target for treatment of neonatal brain injury.

## Materials and Methods

### Cell Culture and Hypoxia Treatment

The PC-12 cells (Kunming Institute of Zoology, Kunming, China) were plated onto flasks at a density of 1 × 10^4^ cells/ml in DMEM with 10% (v/v) fetal bovine serum (FBS), 100 U/ml penicillin and 100 μg/ml streptomycin (all from Invitrogen, Carlsbad, CA, USA). Cultures under normoxia were maintained at 37°C in a humidified incubator containing 95% air and 5% CO_2_. Culture medium was changed every other day. Hypoxia treatment was performed as described previously (Mo et al., 2012). In brief, the PC-12 cells were exposed to an atmosphere composed of 1% O_2_, 94% N_2_ and 5% CO_2_ in an oxygen control incubator (Heal Force, Shanghai, China), and the duration was 6 h.

### Cell Counting Kit-8 (CCK-8) Assay

A Cell Counting Kit-8 (CCK-8) assay (Dojindo Molecular Technologies, Gaithersburg, MD, USA) were utilized for estimation of cell viability. Briefly, after seeding in 96-well plates with 5 × 10^3^ cells per well, cells were cultured and treated differently. Then, the CCK-8 solution was added into each well, followed by incubation for 1 h at 37°C in a humidified atmosphere containing 95% air and 5% CO_2_. The absorbance at 450 nm was measured by using a Microplate Reader (Bio-Rad, Hercules, CA, USA).

### Apoptosis Assay

Flow cytometry analysis following dual staining with Annexin V-FITC and PI was performed to identify and quantify the apoptotic cells. Briefly, the PC-12 cells (1 × 10^5^ cells/well) were plated in six-well plates. Then, treated cells were washed twice with cold PBS and resuspended in binding buffer. Dual staining was performed according to the instructions of an Annexin V-FITC/PI apoptosis detection kit (Beijing Biosea Biotechnology, Beijing, China). Finally, cell apoptosis was measured by a flow cytometer (Beckman Coulter, Miami, FL, USA).

### miRNA Transfection

miR-210 mimic, scramble miRs, miR-210 inhibitor and its negative control, which was referred to as NC, were synthesized by GenePharma Co. (Shanghai, China). On the basis of the supplier’s protocol, cell transfections were conducted using Lipofectamine 3000 reagent (Invitrogen). Because the highest transfection efficiency occurred at 48 h, 72 h post-transfection was considered as the harvest time in the subsequent experiments.

### Stable Transfection

Short-hairpin (sh) RNA directed against BNIP3 or shRNA carrying a non-targeting sequence was sub-cloned into the U6/GFP/Neo plasmid (GenePharma, Shanghai, China) and the resultant plasmids were referred to as sh-BNIP3 or sh-NC. Full-length BNIP3 sequences were ligated into the pcDNA3.1 (Invitrogen) and the resultant plasmid was referred to as pc-BNIP3. The Lipofectamine 3000 reagent was used for cell transfection according to the manufacturer’s instructions. The stably transfected cells were selected using culture medium containing 0.5 mg/ml G418 (Sigma-Aldrich, St. Louis, MO, USA). After approximately 4 weeks, G418-resistant cell clones were established. sh-NC and pcDNA3.1 were respectively transfected into PC-12 cells, acting as negative control of sh-BNIP3 and pc-BNIP3.

### Quantitative Real-Time Polymerase Chain Reaction (qRT-PCR)

Total RNA of cells was extracted using Trizol reagent (Life Technologies Corporation, Carlsbad, CA, USA) following the manufacturer’s instructions. cDNA was synthesized by using the Taqman miR Reverse Transcription Kit and quantitative polymerase chain reaction (PCR) was performed with Taqman Universal Master Mix II (both from Applied Biosystems, Foster City, CA, USA) according to the protocol of suppliers. Meanwhile, Multiscribe RT kit and SYBR Green PCR Master Mix were utilized for reverse transcription of cDNA and quantitative PCR of BNIP3 mRNA and GAPDH, following the instructions of Applied Biosystems. Relative expression was calculated on the basis of the 2^−ΔΔC_t_^ method (Livak and Schmittgen, 2001), normalizing to U6 (miR-210) or GAPDH (BNIP3 mRNA).

### Dual Luciferase Activity Assay

The wild-type BNIP3 3′UTR sequence containing a putative miR-210-binding site, predicted using online TargetScan software^1^ and miRBase database^2^ (Chung et al., 2015), was sub-cloned into pMiR-report vector (Ambion, Austin, TX, USA), and the resultant plasmid was referred to as BNIP3-WT. Mutation at the miR-210-binding site of BNIP3-WT, which was referred to as BNIP3-Mut, was formed using the QuikChange site-directed mutagenesis kit (Stratagene, La Jolla, CA, USA). Cells were co-transfected with BNIP3-WT or BNIP3-Mut and miR-210 mimic or scramble miRs using Lipofectamine 3000 reagent. Reporter assays were done using the dual-luciferase assay system (Promega, Madison, WI, USA) according to the manufacturer’s instructions.

### Migration and Invasion Assay

Cell migration and invasion were determined by using a modified two-chamber migration assay and 24-well Millicell Hanging Cell Culture inserts, respectively. The pore size of membranes was 8 μm, and the insert for cell invasion assay was pre-coated with Matrigel (BD Biosciences, Bedford, MA, USA). Briefly, cells suspended in 200 μl of serum-free medium were seeded on the upper compartment, whereas the lower compartment was filled up with 600 μl of complete medium. After incubation at 37°C, cells were fixed with methanol and non-traversed cells on the upper surface of the filter were carefully removed with a cotton swab. Traversed cells attached on the lower side of the filter were stained with crystal violet and counted microscopically. The data were presented as the average number of cells attached to the bottom surface from five randomly chosen fields.

### Western Blot Analysis

The proteins of treated cells were extracted using RIA lysis buffer (Beyotime Biotechnology, Shanghai, China) with the presence of protease inhibitors (Roche, San Francisco, CA, USA). After quantification using the BCA™ Protein Assay Kit (Pierce, Appleton, WI, USA), the proteins were separated by using a Bio-Rad Bis-Tris Gel system according to the manufacturer’s instructions. Then, proteins in the gels were blotted to polyvinylidene difluoride (PVDF) membranes, followed by blocking with 5% non-fat milk. After rinsing, membranes were incubated at 4°C overnight with primary antibodies against B cell lymphoma-2 (Bcl-2, ab196495), Bcl-2-associated X protein (Bax, ab182733), BNIP3 (ab109362), PI3K (ab40755), phospho-PI3K (p-PI3K, ab191606), mTOR (ab134903), phospho-mTOR (p-mTOR, ab137133), GAPDH (ab128915; all from Abcam, Cambridge, UK), pro caspase-3 (9665), cleaved caspase-3 (9661), pro caspase-9 (9508), cleaved caspase-9 (9507), phospho-AKT (p-AKT, 4060), AKT (4685), p70S6K (9202) or phospho-p70S6K (p-p70S6K, 9206; all from Cell Signaling Technology, Beverly, MA, USA). After rinsing, membranes were incubated with secondary antibodies marked by horseradish peroxidase at room temperature for 1 h. After rinsing, the membranes were transferred into the Bio-Rad ChemiDoc™ XRS system, and the proteins in the membranes were visualized by using Immobilon Western Chemiluminescent HRP Substrate (Millipore, Billerica, MA, USA). The signals and intensity of the bands were quantified using Image Lab™ software (Bio-Rad).

### Statistical Analysis

Each experiment was repeated three times. The results of multiple experiments are presented as mean ± SEM. Statistical analyses were performed using SPSS 19.0 statistical software. The *P*-values were calculated using one-way analysis of variance (ANOVA). A *P*-value of <0.05 was considered to indicate a statistically significant result.

## Results

### Hypoxia Induced Cell Injury

**Figure 1:**
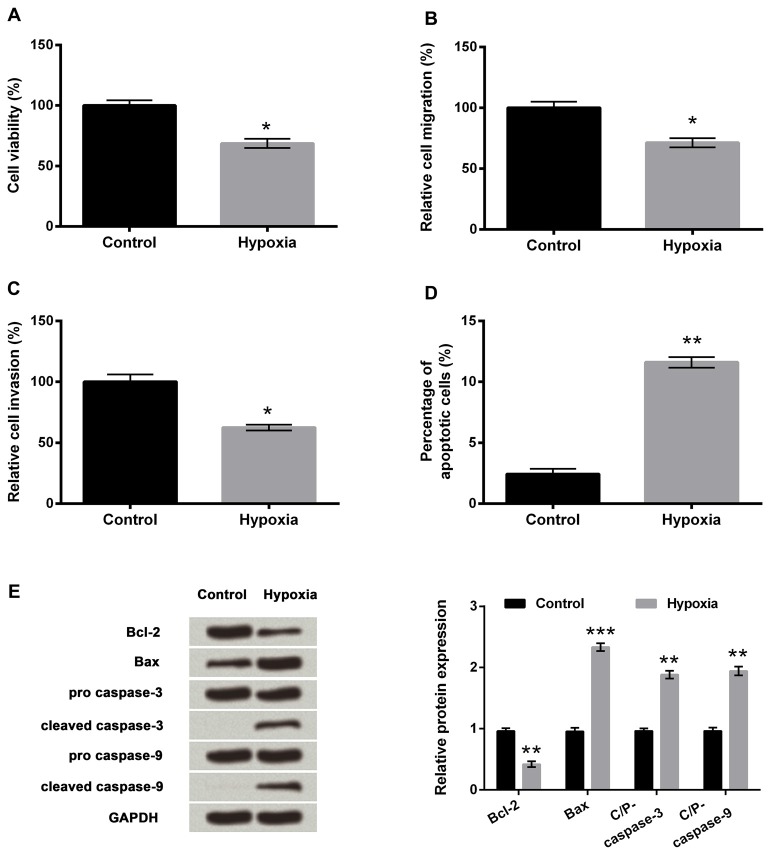
PC-12 cell injury induced by hypoxia. **(A–D)** Effects of hypoxia treatment on cell viability, migration, invasion and apoptosis. **(E)** Western blotting analysis of apoptosis-related factors. Data presented are the mean ± SEM of at least three independent experiments. *P < 0.05; **P < 0.01; ***P < 0.001. Bcl-2, mammalian B cell lymphoma-2; Bax, Bcl-2-associated X protein; C/P-, cleaved/pro.

To investigate the outcomes of hypoxic injury, the PC-12 cells were exposed to hypoxia. Results revealed that hypoxia significantly suppressed cell viability, migration and invasion (Figures 1A–C, *P* < 0.05) compared to a control group of cells and significantly increased the percentage of apoptotic cells (Figure 1D, *P* < 0.01). Western blot analysis revealed markedly reduced expression of Bcl-2 (anti-apoptotic factor, *P* < 0.01), while significantly increased expression of Bax (pro-apoptotic factor, *P* < 0.001) in the hypoxia group compared to the control group of cells (Figure 1E). Meanwhile, cleaved/pro caspase-9 and cleaved/pro caspase-3 were dramatically increased by hypoxia compared with the control group (both *P* < 0.01), indicating the caspase-dependent signaling pathway was activated by hypoxia.

### Hypoxia Up-Regulated miR-210

**Figure 2:**
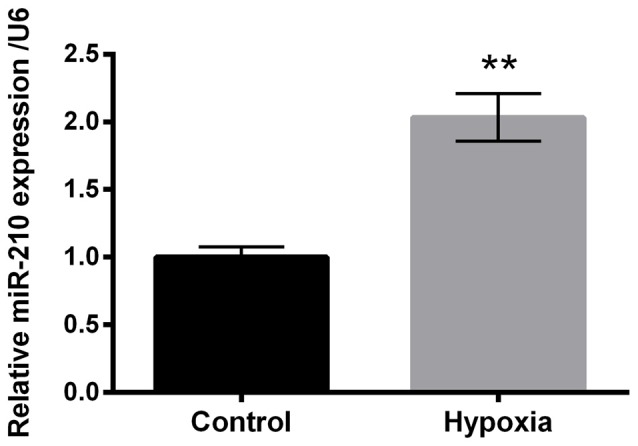
Expression of microRNA (miR)-210 in hypoxia-treated PC-12 cells. Data presented are the mean ± SEM of at least three independent experiments. **P < 0.01.

To investigate the expression of miR-210 in hypoxia-treated PC-12 cells, quantitative real-time PCR (qRT-PCR) was carried out. The results revealed significant up-regulation of miR-210 expression levels in hypoxia-treated PC-12 cells compared to control group of cells (*P* < 0.01, Figure 2).

### Expression of miR-210 Was Overexpressed or Suppressed by Respective Mimic or Inhibitor Transfection

**Figure 3:**
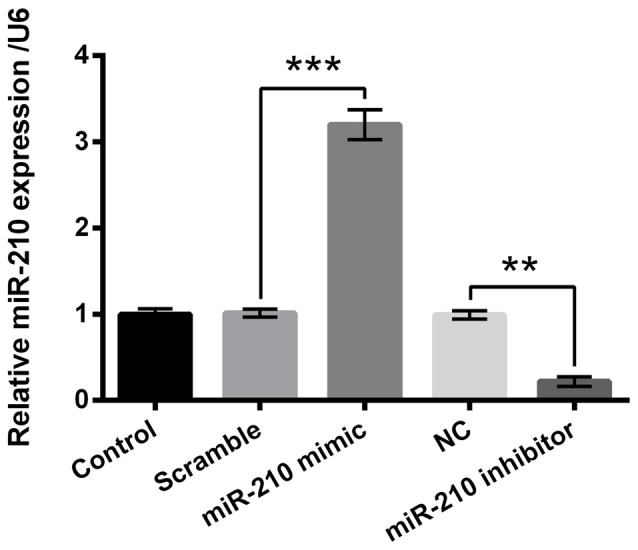
Overexpression or suppression of microRNA (miR)-210 by mimic or inhibitor transfection. Data presented are the mean ± SEM of at least three independent experiments. **P < 0.01; ***P < 0.001. NC, negative control of miR-210 inhibitor.

PC-12 cells were transfected with miR-210 mimic, miR-210 inhibitor or their negative controls. Figure 3 demonstrated that the expression level of miR-210 in the miR-210 mimic group was significantly higher than the scramble group of cells (*P* < 0.001), while there was significant decrease in the expression level of miR-210 in the miR-210 inhibitor group compared to the NC group of cells (*P* < 0.01). These results suggested that miR-210 was suppressed or overexpressed by treatment with miR-210 inhibitor or miR-210 mimic, respectively.

### miR-210 Overexpression Alleviated the Injury Induced by Hypoxia in PC-12 Cells

**Figure 4:**
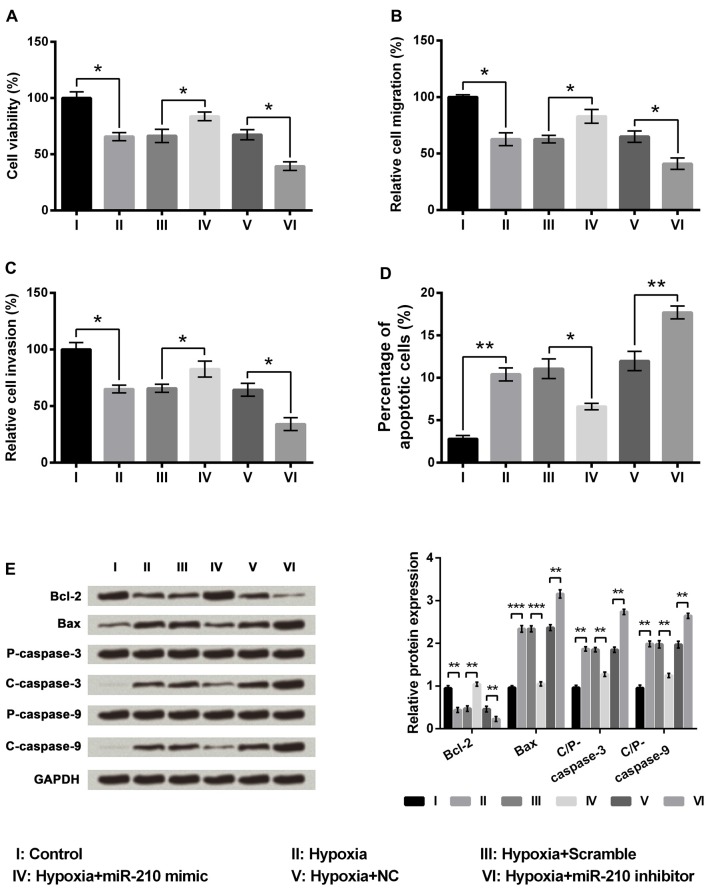
MicroRNA (miR)-210 overexpression alleviated the injury induced by hypoxia in PC-12 cells. **(A–D)** Overexpression of miR-210 increased cell viability, cell migration, cell invasion, and decreased cell apoptosis. **(E)** Western blotting analysis of apoptosis related factors. Data presented are the mean ± SEM of at least three independent experiments. *P < 0.05; **P < 0.01; ***P < 0.001. Bcl-2, mammalian B cell lymphoma-2; Bax, Bcl-2-associated X protein; C/P-, cleaved/pro; NC, negative control of miR-210 inhibitor.

We then determined whether miR-210 regulated the injury induced by hypoxia in PC-12 cells. A marked increase in PC-12 cell viability was caused by miR-210 mimics compared to the scramble group of cells (Figure 4A, *P* < 0.05), while cell viability was significantly decreased following suppression of miR-210 expression as in the hypoxia+miR-210 inhibitor group compared to the hypoxia+NC group (*P* < 0.05). Similar results were obtained in migration and invasion assays (Figures 4B,C). The percentage of apoptotic cells was decreased significantly in the hypoxia+miR-210 mimic group as compared to the scramble group (*P* < 0.05) while it was increased markedly in the hypoxia+miR-210 inhibitor group compared to hypoxia+NC group (Figure 4D, *P* < 0.01). Western blot results of apoptosis related factors showed Bcl-2 was significantly up-regulated while Bax, cleaved/pro caspase-9 and cleaved/pro caspase-3 were all down-regulated in the hypoxia+miR-210 mimic group compared to the hypoxia+scramble group (Figure 4E, *P* < 0.01 or *P* < 0.001). Cell proliferation, migration and invasion were inhibited, but apoptosis was promoted, due to hypoxia, could be alleviated by the overexpression of miR-210, while it could be aggravated by miR-210 suppression.

### miR-210 Targeted BNIP3

**Figure 5:**
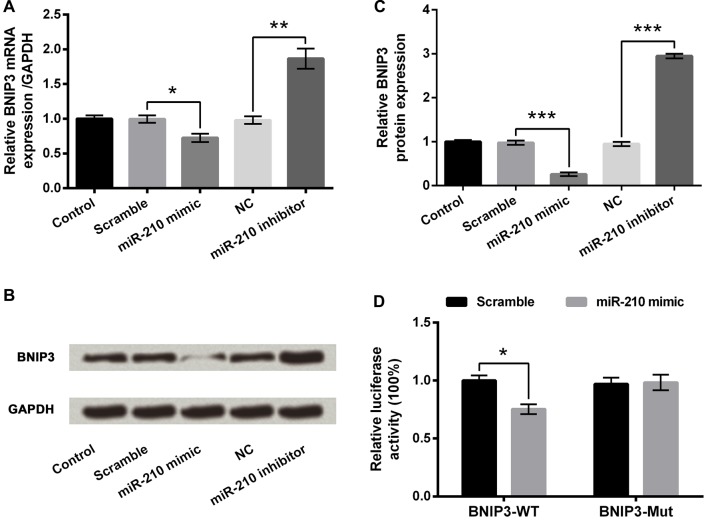
BNIP3 is a target of miR-210. **(A)** mRNA expression of BNIP3. **(B,C)** Protein expression of BNIP3. **(D)** Dual luciferase reporter assay. Data presented are the mean ± SEM of at least three independent experiments. *P < 0.05; **P < 0.01; ***P < 0.001. BNIP3, Bcl-2 adenovirus E1B 19 kDa-interacting protein 3; NC, negative control of miR-210 inhibitor; BNIP3-WT, pMiR-report vector carrying wild-type BNIP3 3′-untranslated region (3′UTR) containing miR-210-binding sites; BNIP3-Mut, mutant BNIP3-WT.

The involvements of miR-210 overexpression, induced by hypoxia, in the regulation of BNIP3 expression in PC-12 cells were investigated. First of all, the nucleotide coding region of BNIP3 mRNA was analyzed. Screen utilizing online TargetScan software and miRBase database revealed the putative binding between miR-210 and BNIP3 3′UTR. Subsequent experiments showed that both mRNA and protein expression of BNIP3 were significantly suppressed (*P* < 0.05 or *P* < 0.001) in the miR-210 mimic group of cells, and were significantly increased (*P* < 0.01 or *P* < 0.001) in the miR-210 inhibitor group of cells (Figures 5A–C). In addition, co-transfection of BNIP3-WT and miR-210 mimic showed significant down-regulation of luciferase activity compared with co-transfection of BNIP3-WT and scramble miRs (Figure 5D, *P* < 0.05), whereas comparison between co-transfections with BNIP3-Mut was non-significant, supporting that the miR-210 mimic could directly bind to BNIP3 3′UTR. Hence, BNIP3 was a target of miR-210.

### miR-210 Functioned by Down-Regulating BNIP3

**Figure 6:**
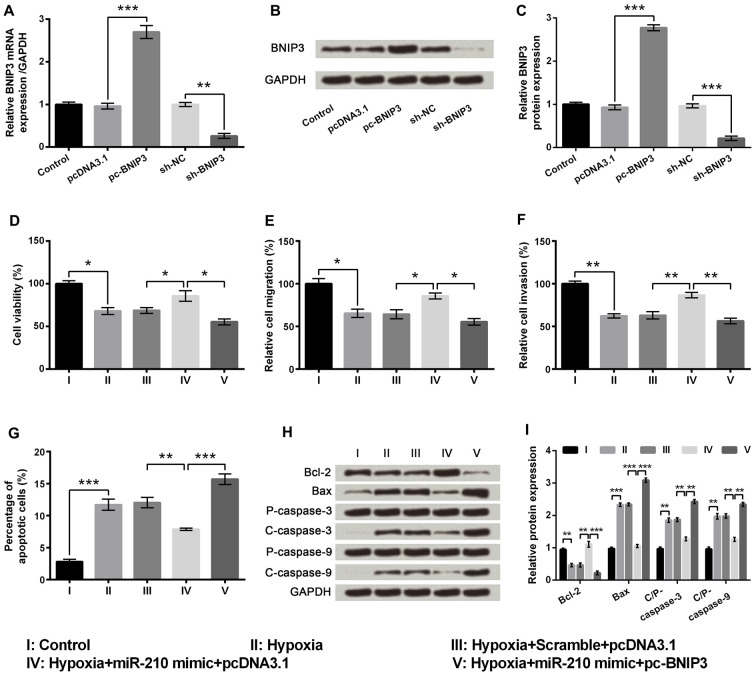
MicroRNA (miR)-210 overexpression ameliorated hypoxia-induced injury in PC-12 cells by down-regulating BNIP3 expression. **(A)** Aberrant mRNA expression of BNIP3 in PC-12 cells. **(B,C)** Aberrant protein expression of BNIP3 in PC-12 cells. **(D–G)** Effects of miR-210 overexpression on cell viability, migration, invasion and apoptosis in hypoxia-treated PC-12 cells were reversed by up-regulating BNIP3. **(H,I)** Western blotting analysis of apoptosis related factors. Data presented are the mean ± SEM of at least three independent experiments. *P < 0.05; **P < 0.01; ***P < 0.001. BNIP3, Bcl-2 adenovirus E1B 19 kDa-interacting protein 3; Bcl-2, mammalian B cell lymphoma-2; Bax, Bcl-2-associated X protein; C/P-, cleaved/pro; sh-NC, U6/GFP/Neo plasmid carrying a non-targeting sequence; sh-BNIP3, U6/GFP/Neo plasmid carrying sh RNA against BNIP3; pc-BNIP3, pcDNA3.1 carrying the full-length of BNIP3.

There was a significant increase in the mRNA and protein expression levels of BNIP3 in the pc-BNIP3 group of cells compared to the pcDNA3.1 group (*P* < 0.001), whereas there was a significant decrease in the mRNA and protein expression level of BNIP3 in the sh-BNIP3 group of cells compared to the sh-NC group (Figures 6A–C, *P* < 0.01 or *P* < 0.001). Subsequent experiments demonstrated significant decreases of cell viability (Figure 6D, *P* < 0.05), migration (Figure 6E, *P* < 0.05) and invasion (Figure 6F, *P* < 0.01) as well as marked increase of cell apoptosis (Figure 6G, *P* < 0.001) in the hypoxia+miR-210 mimic+pc-BNIP3 group of cells compared to hypoxia+miR-210 mimic+pcDNA3.1 group of cells. In addition, Bcl-2 was remarkably down-regulated (*P* < 0.001) while Bax, cleaved/pro caspase-9 and cleaved/pro caspase-3 were significantly up-regulated (*P* < 0.01) in the hypoxia+miR-210 mimic+pc-BNIP3 group of cells compared to the hypoxia+miR-210 mimic+pcDNA3.1 group of cells (Figures 6H,I). Results suggested that miR-210 overexpression alleviated hypoxia-induced PC-12 cell injury by down-regulating BNIP3 expression.

### BNIP3 Inactivated the PI3K/AKT/mTOR Pathway

**Figure 7:**
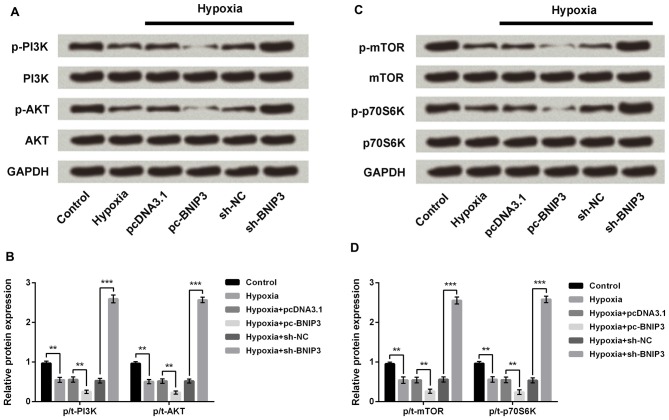
BNIP3 affected the hypoxia-induced inhibition of the PI3K/AKT/mTOR signal pathway. **(A,B)** Western blotting analysis of factors associated with the PI3K/AKT pathway. **(C,D)** Western blotting analysis of factors associated with the mTOR pathway. Data presented are the mean ± SEM of at least three independent experiments. **P < 0.01; ***P < 0.001. BNIP3, Bcl-2 adenovirus E1B 19 kDa-interacting protein 3; sh-NC, U6/GFP/Neo plasmid carrying a non-targeting sequence; sh-BNIP3, U6/GFP/Neo plasmid carrying sh RNA against BNIP3; pc-BNIP3, pcDNA3.1 carrying the full-length of BNIP3; PI3K, phosphatidylinositol 3-kinase; mTOR, mammalian target of rapamycin.

**Figure 8:**
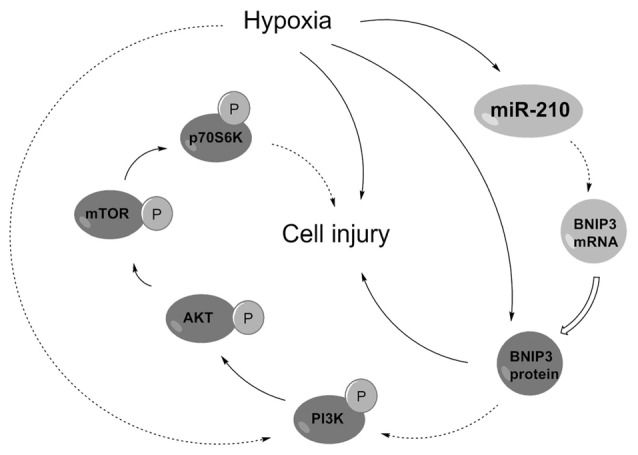
Regulatory circuit of the microRNA (miR)-210-associated modulation in PC-12 cells under hypoxia. Solid arrows indicated positive regulation but dotted indicated negative regulation. BNIP3, Bcl-2 adenovirus E1B 19 kDa-interacting protein 3; PI3K, phosphatidylinositol 3-kinase; mTOR, mammalian target of rapamycin.

PI3K/AKT/mTOR has been reported as an essential pathway that participates in the neuroprotective effect of brain-derived neurotrophic factor, indicating the crucial role of this pathway in neuroprotection (Chen et al., 2013). Western blotting analysis demonstrated the phosphorylated levels of key kinases in the PI3K/AKT/mTOR signaling pathway, including PI3K, AKT, mTOR and p70S6K, were all dramatically decreased by hypoxia (Figures 7A–D, *P* < 0.01). Moreover, the hypoxia-induced decreases were further decreased by BNIP3 overexpression (*P* < 0.01), but were reversed by BNIP3 knockdown (*P* < 0.001). These results suggested that hypoxia-induced inhibition of the PI3K/AKT/mTOR signal pathway was further inhibited by BNIP3 overexpression. Taken together, the schematical summarization of all the results was shown in Figure 8.

## Discussion

The data presented in this study revealed that miR-210 was overexpressed in hypoxia-induced PC-12 cells. The underlying molecular mechanism for miR-210 in regulating cell proliferation, apoptosis and migration was complicated, as miR-210 overexpression protected PC-12 cells against hypoxia-induced injury via suppression of BNIP3 expression, involving the PI3K/AKT/mTOR signal pathway. This study may provide a theoretical basis for this disease molecular mechanism and suggest a new treatment approach for neonatal brain injury.

Huang et al. (2009) once identified the promoter region of miR-210 contains a functional hypoxia-responsive element (HRE). As a target of HIF-1α, miR-210 is the only miR that is consistently up-regulated in normal and transformed cells, which are exposed to hypoxia, in all the literatures reported previously (Biswas et al., 2010; Chen et al., 2010). Previous studies demonstrated that miR-210 was up-regulated in NPCs cultured under hypoxic conditions (Wang et al., 2013). Also, hypoxic conditions had been reported to promote the NPC proliferation in both *in vitro* and *in vivo* models (Zhu et al., 2005; Zhao et al., 2008). Similar to these results, our study demonstrated the abnormal expression of miR-210 in PC-12 cells under hypoxia.

Mounting evidence supports diverse functions of miR-210 in multiple biological processes associated with hypoxia, for example, cell cycle, proliferation, angiogenesis, apoptosis, DNA damage repair, mitochondrial metabolism, differentiation and tumor growth. Cell apoptosis of HeLa cells has been proved to be elevated by the miR-210 blockade (Cheng et al., 2005). A recent study has reported cell survival and autophagy were promoted by miR-210 (Xu et al., 2016). In our study, hypoxia induced decreases of cell viability, migration and invasion as well as increased apoptosis. The activated mitochondrial- and caspase-dependent pathways, induced by hypoxia, partially explained the alteration of cell survival and apoptosis. In addition, we interestingly figured out that miR-210 mimics could ameliorate hypoxia-induced cell injury of PC-12 cells, whereas silencing of miR-210 expression leads to the opposite outcome. The results suggested a possible cytoprotective effect of miR-210 on survival of brain cells.

To understand the miR mechanisms in controlling cellular behavior, we used a Bioinformatics method to virtually screen the possible targets of miR-210. In our study, we focused on the interaction between BNIP3 and miR-210, presenting that BNIP3 could be negatively regulated by miR-210. Additional luciferase reporter assays further established that the BNIP3 3′UTR was recognized by miR-210. In diverse cell types under hypoxic conditions, mRNA and protein expressions of BNIP3 are prominently up-regulated (Bruick, 2000), indicating that, in hypoxia-treated cells, BNIP3 is transcriptionally modulated by the HIF complex. Moreover, BNIP3 is indicated to function as a pro-apoptotic BH3-only protein, which is related to the pathogenesis of many diseases, such as cancer and cardiovascular disease (Wang et al., 2013). Recent studies demonstrated that BNIP3 induces mitochondrial turnover and autophagy, resulting in cell death (Lee et al., 2011; Rikka et al., 2011). Results in our study found the effects of miR-210 overexpression on cell viability, migration, invasion and apoptosis were all reversed by BNIP3 overexpression in hypoxia-treated PC-12 cells, suggesting miR-210 functioned through negative regulation of BNIP3. This study is the first to report BNIP3 as a target of miR-210 in PC-12 cells under severe hypoxia, which was consistent with that reported in NPCs previously. Considering that both miR-210 and BNIP3 are pivotal targets of HIF-1, the discovery of miR-210-BNIP3 contributes to the regulatory network of HIF-1 under hypoxia in PC-12 cell, and the network in other cell types needs more experiments.

Carloni et al. (2010) has reported that the neuroprotective effect of rapamycin in neonatal hypoxia-ischemia is likely associated with the up-regulation of autophagy and activation of the PI3K-AKT-mTOR pathway. In both the PC-12 cell models of oxygen glucose deprivation and the rat brain model of permanent focal ischemia, ischemic preconditioning induced the activation of autophagy, demonstrating that the generation and degradation of autophagosomes was increased after ischemic reperfusion (Park et al., 2009). Our study demonstrated that aberrantly expressed BNIP3, which might be modulated by miR-210, could affect the activation of the PI3K/AKT/mTOR signaling pathway in PC-12 cells under hypoxia. Results suggested that miR-210 overexpression alleviated the injury of PC-12 cells induced by hypoxia via suppressing BNIP3 expression and activating the PI3K/AKT/mTOR signal pathway. These mechanisms may underlie the development and progression of neonatal brain injury. Although the interaction between miR-210 and BNIP3 has been reported in the hypoxia-treated NPC cells previously, we verified the links between miR-210 and BNIP3 in another neural cell line, PC-12 cells. Moreover, the downstream signaling pathways besides the interaction between miR-210 and BNIP3 in hypoxia-treated cells are reported for the first time.

In summary, we report that miR-210 protect PC-12 cells against hypoxia-induced injury by targeting BNIP3. The suppression of BNIP3, directly regulated by miR-210 overexpression, may partially contribute to the protection against hypoxic injury in PC-12 cells.

## Author Contributions

In this study, YL designed the work, drafted the manuscript, conducted the experiments and contributed to interpretation of data. XZ and YZ conducted the experiments, analyzed the data and helped to prepare the manuscript. YD helped to prepare the manuscript and images, collected and analyzed the data and literature. All authors read and approved the final manuscript. All authors agreed to be accountable for all aspects of the study in ensuring that questions related to the accuracy or integrity of any part of the work are appropriately investigated and resolved.

## Conflict of Interest Statement

The authors declare that the research was conducted in the absence of any commercial or financial relationships that could be construed as a potential conflict of interest.

## Footnotes

^1^http://www.targetscan.org/vert_71

^2^http://www.mirbase.org

## Abbreviations

Bax: Bcl-2-associated X protein
Bcl-2: B cell lymphoma-2
BNIP3: Bcl-2 adenovirus E1B 19 kDa-interacting protein 3
CCK-8: Cell Counting Kit-8
FBS: fetal bovine serum
HI: hypoxic ischemic
HIF-1α: hypoxia inducible factor-1α
miR: microRNA
NPC: neural progenitor cells
PI3K/AKT/mTOR: Phosphatidylinositol 3-kinase/AKT/mammalian target of rapamycin, 3′UTR, 3′-untranslated region.
